# Endothelin-3 Suppresses Luteinizing Hormone Receptor Expression by Regulating the cAMP-PKA Pathway in Hen Granulosa Cells

**DOI:** 10.3390/cimb46080464

**Published:** 2024-07-23

**Authors:** Yurong Tai, Deping Han, Xue Yang, Ganxian Cai, Huaiyu Li, Junying Li, Xuemei Deng

**Affiliations:** 1 Sanya Institute, China Agricultural University, Sanya, 572000, China; tyd1992@126.com (Y.T.);; 2 Hainan Seed Industry Laboratory, Yazhou 572024, China; 3 Beijing Key Laboratory for Animal Genetic Improvement, National Engineering Laboratory For Animal Breeding, China Agricultural University, Beijing 100000, Chinacgx@cau.edu.cn (G.C.);

**Keywords:** Silky Fowl, EDN3, granulosa cell, LH, cAMP

## Abstract

Previous research identified the expression of *EDN3* in granulosa cells of preovulatory follicles in chickens. Notably, the expression level of *EDN3* in Silky Fowl with low egg-laying performance was significantly higher than that in high-yield laying breed White Leghorn. Given the crucial role of granulosa cells in follicular development and maturation, it is very important to study the effect of *EDN3* on the biological function of granular cells. In this study, an *EDN3* overexpression plasmid was constructed and transfected into granular cells. The viability of these cells was detected using quantiative (qPCR), Cell Counting Kit-8 (CCK8), and 5-Ethynyl-2′-deoxyuridine (EdU) assays. Gonadal hormone synthesis was detected using enzyme-linked immunosorbent assay (ELISA) techniques. Finally, transcriptome sequencing was employed to identify differentially expressed genes. Result showed thatoverexpression of *EDN3* was observed to promote cell viability. In addition, it significantly inhibits the expressions of *LHR* and cAMP-PKA signaling pathways. Cell transcriptome sequencing data displayed that *EDN3* can upregulate energy metabolism and immune-related signaling pathways, whereas follicle maturation and the GnRH signaling pathway were downregulated. In conclusion, this study demonstrates that *EDN3* can enhance granulosa cell viability and inhibit the expression of *LHCGR*, a process likely mediated through the cAMP-PKA signaling pathway. However, further evidence is required to substantiate the regulatory relationship between *EDN3* and the cAMP-PKA signaling pathway.

## 1. Introduction

Endothelins derived from residual vasoconstrictive peptides have been isolated and are known to be highly powerful vasoconstrictor peptides [1,2]. The three endothelin peptides with vasoconstrictive activity were named endothelin-1 (*EDN1*; ET-1), endothelin-2 (*EDN2*; ET-2), and endothelin-3 (*EDN3*; ET-3) and have been found in humans and other mammals [2]. These three EDNs are biologically active peptides of 21 amino acids encoded by different genes [3]. Each EDN originated from a substantial precursor containing approximately 200 amino acids. The precursor is decomposed by big EDN, a non-biologically active intermediate product of approximately 38 amino acids. The endothelin-converting enzyme (ECE) cleaves large EDN into biologically active EDN peptides [3].

EDNs and endothelin receptors (EDNRs) regulate many physiological and pathological processes, such as cardiovascular development and function, pulmonary hypertension, proliferation, differentiation, migration of neural crest cells (NCCs), and pigmentation formation [4,5,6]. In blood vessels, *EDN1* is mainly expressed in endothelial cells, which can induce vasoconstriction through *EDNRA* expression on vascular smooth muscle cells [7]. In ovaries, *EDN2* is mainly expressed in granulosa cells (GCs) and may mediate ovulation via *EDNRA*. *EDN3* is expressed in the surface ectoderm and intestinal mesenchyma of embryos [8,9]. It may induce the proliferation, migration, and differentiation of trunk NCCs and their derivatives, such as melanocytes, and may thus be one of the key signals of pigmentation and ganglionopathy [4,10]. In poultry, *EDN3* was found to play a role in NCCs differentiation, survival, and proliferation in vitro. The long-term addition of exogenous *EDN3* can induce melanocyte differentiation in poultry NCCs, resulting in a large increase in the number of melanocytes [11,12]. Adding *EDN3* during NCCs culture in quails is necessary to maintain cell viability and promote cell proliferation [12]. Therefore, the effect of *EDN3* on cell growth and viability in germ cells is also worth exploring.

Endothelin has also been depicted to affect gonadal hormone production in rat, pig, human, and bovine ovarian cells [13]. In rat GC cultures, adding *EDN1* and *EDN3* decreased basal estrogen secretion [14]. In a human yellow body cell culture, *EDN1* significantly reduced basal- and HCG-induced progesterone secretion, but *EDN3* had no major effect [15]. *EDN2* is expressed in bovine GCs and early luteal development cells. LH can induce the expression of *EDN2* mRNA in bovine GCs [16]. Few studies have investigated the effects of *EDN3* on ovaries. No significant expressed levels of *EDN3* were detected in human ovaries [17], and very few were detected in whole mouse ovary samples [18]. Hence, it is very interesting to study the effect of *EDN3* on the synthesis of gonadal hormone in poultry germ cells. Silky Fowl (SF) is an ideal model for *EDN3* gene overexpression. The *EDN3* gene region of this chicken breed has undergone chromosomal inversion and duplication mutations, resulting in an abnormally high expression of the *EDN3* gene. The previous transcriptome sequencing of our research disclosed that the expression of *EDN3* SF GCswas significantly higher than that of White Leghorn (WL) [19]. Therefore, this study was conducted to examine the effects of *EDN3* on the growth and synthesis of the gonadal hormone of GCs. This represents the initial investigation into the function of the EDN3 gene in hens.

## 2. Materials and Methods

### 2.1. Animals and Sample Collection

Laying WL and SF were used in the present study. They were raised under natural light and temperature conditions at the Poultry Breeding Experimental Farm at China Agricultural University (Beijing, China) and were provided unlimited food and water. The hens were euthanized by cervical dislocation before ovary tissue collection for culture and transcriptome sequencing. Healthy hens, aged 210 days (peak laying period) and with regular laying sequences, were selected. The hens were killed by cervical dislocation 2 h after oviposition. All ovarian follicles were collected from the abdominal cavity under aseptic conditions and placed in phosphate-buffered saline PBS (pH 7.4). We selected preovulatory follicles (F1–F5) and separated and cultured GCsbased on the previously described methods [20].

### 2.2. Construction and Identification of pcDNA3.1-EDN3 Vectors

Total RNA was isolated from the GC tissue of SF using TRIzol reagent (Takara, Kyoto, Japan), according to the manufacturer’s instructions. RNA quality and concentration were determined using agarose gel electrophoresis and NanoDrop 2000 spectrophotometry (Thermo Fisher Scientific, Carlsbad, CA, USA). In total, 1 µg of total RNA was reverse transcribed to cDNA using a FastKing gDNA Dispelling RT SuperMix (TIANGEN, Beijing, China), with 4.0 μL of 5 × FastKing-RT SuperMix (TIANGEN, Beijing, China), total RNA volume calculated by standard, RNase-free H_2_O was added to 10 μL. Thermal cycling was performed for 15 min at 42 °C and then 3 min at 95 °C. RT products were used as templates for PCR. Primers (Table 1) were synthesized by Sangon Biotech Co., Ltd. (Shanghai, China). To clone the CDS region of EDN3, a PCR mixture with a total volume of 20 μL was prepared, including 1 μL of cDNA, 0.5 μL of up- and downstream primers, 1 μL of PrimeSTAR Max Premix (Takara, Kyoto, Japan), and 7 μL of dd H_2_O. The PCR reaction was performed at 98 °C for 3 min, followed by 30 cycles (98 °C for 10 s, 60 °C for 10 s, and 72 °C for 5 s), and ending at 72 °C for 5 min. The PCR products were detected using 1.5% agarose gel electrophoresis.

To construct the overexpression vector, an OMAGE glue recovery kit (Omega Bio-Tek, Norcross, GA, USA) was used to purify the PCR product. The vector pcDNA3.1-EGFP was then incised with two enzymes: restriction endonuclease NheI and BamHI (New England Biolabs, NEB, Ipswich, MA, USA). The total enzyme digestion system contained 1 μg of pcDNA3.1-EGFP in 40 μL, 1 μL of NheI, 1 μL of BamHI, and dd H_2_O replenished to 40 μL. The gel recovery product was linked to the plasmid using the SE seamless cloning method and assembly kit (Zhuangmeng Biotechnology Co., LTD., Beijing, China). The recombinant reaction system consisted of 10 μL, containing 50 μg of pcDNA3.1-EGFP, 1 μL of DNA fragment, 1 μL of SE recombinase, 2 μL of 5 × SE clonal buffer, and ddH_2_O supplemented to 10 μL. The reaction mixture was mixed and incubated at 37 °C for 2 h. The pcDNA3.1-EDN3 plasmid was transformed into the Trans5α receptor cells (TransGen Biotech, Beijing, China). Transformed receptive cells were evenly coated in LB Petri dishes, and single colonies were selected and sequenced by the Beijing Genomics Institute (Shenzhen, Guangdong, China). The correct plasmids were extracted.

### 2.3. Transfection of pcDNA3.1-EDN3 Plasmids

GCs were implanted in 12-well plates, and transfection tests were performed when the cells reached 80% density. The mixture of the Liposomes 2000 (Invitrogen, Carlsbad, CA, USA) and OPTI MEM (Invitrogen, Carlsbad, CA, USA) was employed to transfect the plasmid of pcDNA3.1-EDN3 and pcDNA3.1-EGFP (as a control). After 6 h, the liquid was discarded, and a fresh M199 medium (Gibco, Thermo Fisher Scientific, CA, USA) was added. The cells were collected after culturing for 24 h. Overexpression and control experiments were performed thrice. After transfection with the plasmid, qPCR was used to detect EDN3 mRNA expression.

### 2.4. CCK8 Assay

A total of 2 × 10^4^ GCs were added to each well of 96-well microplates (Corning Incorporated, Corning, NY, USA). After the cells were fully attached, they were transfected with pcDNA3.1-EDN3 and pcDNA3.1-EGFP plasmids. Cell viability was analyzed using the cell counting kit-8 (CCK8, Beyotime, Shanghai, China) according to the manufacturer’s protocol. After 6 h, 10 μL of CCK-8 reagent was added to each well and then cultured for 2 h. The cell viability was measured from 1 to 7 days, with six repetitions at each time point. The absorbance was then measured using a microplate analyzer at 450 nm.

### 2.5. EDU Assay

5-Ethynyl-2′-deoxyuridine (EdU, Beyotime, Shanghai, China) staining was used to analyze the GC proliferation status after *EDN3* overexpression. The plasmid was transfected into GCs for 6 h and incubated with 10 μM EdU for 2 h. The cells were fixed with 4% paraformaldehyde for 30 min. Hoechst (Beyotime, Shanghai, China) was used to stain the nuclei. The overexpression and control groups were analyzed under a fluorescence microscope, and the percentage of EdU-positive cells was calculated.

### 2.6. ELISA Assay

The culture supernatant was collected from the cultured cell wells, centrifuged gently, and used for further experiments. The levels of follicle-stimulating hormone (FSH), luteinizing hormone (LH), and progesterone (P4) secreted by GCs in the culture supernatant were detected using an ELISA kit (Shanghai Enzyme-linked Biotechnology Co., Ltd., Shanghai, China), according to the manufacturer’s instructions. Standards and samples were added to the ELISA plate wells. Horseradish peroxidase (HRP)-labeled antigen and detection antibodies were first added to the reaction mixture. The plates were washed after a 60 min incubation at 37 °C. The enzyme-linked working solution was added, and the mixture was incubated for 15 min. The reaction was terminated using a stop solution, and the OD450 value was determined.

### 2.7. Quantitative Real-Time PCR Validation

Total RNA isolated from follicle GC tissue was extracted using TRIzol reagent (Invitrogen, Carlsbad, CA, USA), following the manufacturer’s instructions. PcDNA3.1-EDN3 was used as the experimental group, and pcDNA3.1-EGFP was used as the control group (*n* = 3). A total of 1 μg RNA was reverse transcribed into cDNA using the Fast Quant RT Kit (with gDNase) (Tiangen Biotech Co., LTD, Beijing, China) according to the manufacturer’s instructions. Gene expression levels were quantified by qPCR using SYBR Green Real-time PCR Master Mix (Tiangen Biotech Co., LTD, Beijing, China). The primers for qPCR were designed using Primer Premier 5 software (PREMIER Biosoft, Palo Alto, CA, USA) and were subsequently synthesized (Sangon Biotech Co., LTD, Beijing, China). The primer sequences are listed in Table 2. The cycling parameters used for qPCR amplification were as follows: initial heat denaturation at 95 °C for 4 min; 40 cycles of 95 °C for 30 s, 55–60 °C for 30 s, and 72 °C for 30 s; and a final extension at 72 °C for 5 min. A melting curve analysis was performed to exclude genomic DNA contamination and to confirm primer specificities. Gene expression was normalized using the 2^−∆∆CT^ method with the glyceraldehyde 3-phosphate dehydrogenase (*GAPDH*) gene as an internal standard. Each biological duplicate was controlled using three technical replicates.

### 2.8. RNA Sequencing and Data Analysis

The TruSeq Stranded mRNA LT Sample Prep Kit (Illumina, San Diego, CA, USA) was used to build transcriptome libraries according to the manufacturer’s instructions. Transcriptome sequencing and analysis were conducted by OE Biotech Co., Ltd. (Shanghai, China). The libraries were sequenced on an Illumina Novaseq 6000 platform, generating 150 bp paired-end reads. Approximately 13,828 raw reads were generated for each sample. The raw reads in fastq format were first processed using fastp [21], and low-quality reads were removed to obtain clean reads.

Clean reads were mapped to the Gallus gallus genome website (https://ftp.ensembl.org/pub/release-74/fasta/gallus_gallus/dna/, accessed on 1 July 2023) using HISAT2 [22]. The FPKM [23] of each gene was calculated, and the read counts of each gene were obtained using HTSeq count [24]. PCA analysis was performed using R (v. 3.2.0) to evaluate the biological duplication of the samples.

Differential expression analysis was performed using DESeq2 [25]. A Q value < 0.05 and fold change > 2 or fold change < 0.5 were set as the threshold for significantly differentially expressed genes (DEGs). Hierarchical cluster analysis of DEGs was performed using R (v 3.2.0) to demonstrate the expression patterns of genes in the different groups and samples. A radar map of the top 30 genes was drawn to show the expression of up-regulated or downregulated DEGs using R packet ggradar.

Based on the hypergeometric distribution, an enrichment analysis of the GO [26], KEGG [27], Reactome, and WikiPathways enrichment analysis of DEGs was performed to screen the significantly enriched terms using R (v 3.2.0). R (v 3.2.0) was used to draw the column, chord, and bubble diagrams of significant enrichment terms.

### 2.9. Statistical Analyses

All statistical analyses were performed using Prism 6.0 (GraphPad Software). Data are expressed as means ± standard error of the mean (SEM). Statistical significance was evaluated using Student’s *t*-test or one-way ANOVA. Asterisk coding is indicated in the figure legends as * *p* < 0.05. ** *p* < 0.01. *** *p* < 0.001.

## 3. Results

### 3.1. EDN3 Gene Is Highly Expressed in SF poGCs Tissue

**Figure 1:**
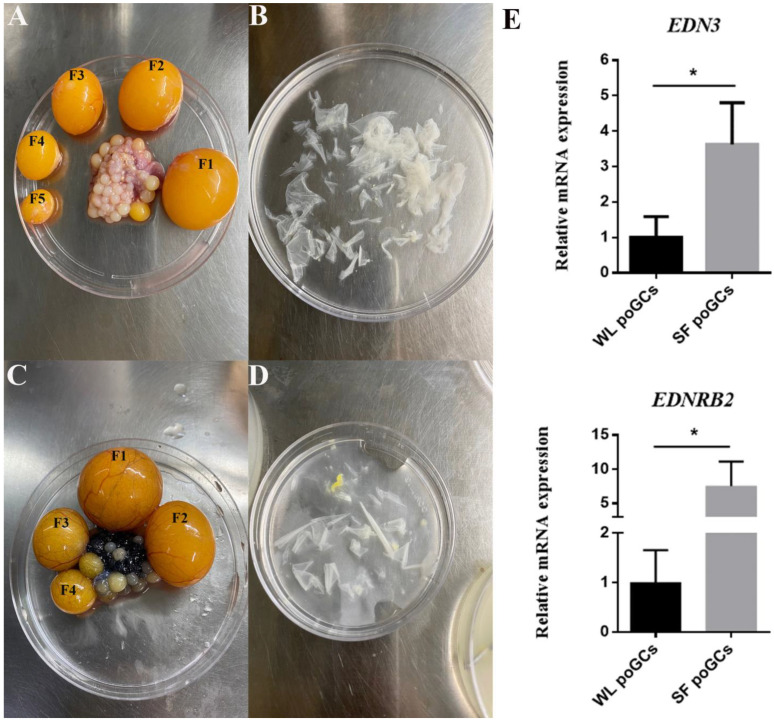
Differential expression of *EDN3* and *EDNRB2* in WL and SF poGCs. (**A**) Morphology of WL (210 days old) preovulation follicle (F1–F5). (**B**) WL poGC tissue separate from stages F1-F5 follicle. (**C)** Morphology of SF (210 days old) preovulation follicle (F1–F4). (**D**) SF poGC tissue separate from stages F1–F4 follicle. (**E**) Differential expression of *EDN3* and *EDNRB2* compared between WL poGCs and SF poGCs (*n = 3*). Data are presented as the mean ± standard deviation (SD). * *p* < 0.05.

Our previous transcriptome sequencing data demonstrated that *EDN3* and its receptor *EDNRB2* were expressed in follicular granulosa cells of WL and SF [19]. In this study, follicular granulosa cells from SF and WL were isolated and cultured (Figure 1A–D). The expressions of *EDN3* and *EDNRB2* were compared by qPCR and found to be significantly higher in the follicular GCs of SF than in those of WL (Figure 1E).

### 3.2. EDN3 Overexpression in WL poGC by Constructing Recombinant Plasmid pcDNA3.1-EDN3

**Figure 2:**
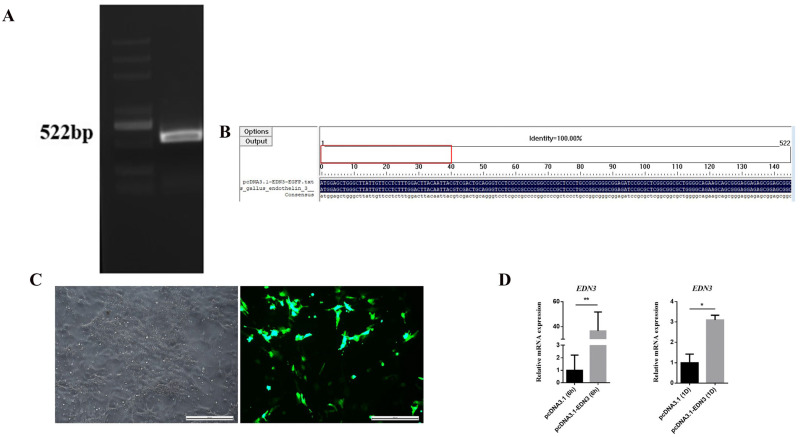
Transfection and validation of pcDNA3.1-EDN3 plasmid. (**A**) PCR amplification of the CDS region of the *EDN3* gene. (**B**) *EDN3* insertion sequence alignment results. (**C**) Expression of fluorescent proteins after pcDNA3.1-EDN3-EGFP transfection. Bar: 200 μm. (**D**) qPCR validate the overexpression of *EDN3* (pcDNA3.1 as a control, *n* = 3). Data are presented as the mean ± standard deviation (SD). * *p* < 0.05. ** *p* < 0.01.

To investigate the effect of *EDN3* on GCs, RNA from SF poGCs was used as a template to amplify the *EDN3* cDNA. The amplified cDNA had the same size as the target gene fragment (522 bp) (Figure 2A). The *EDN3* cDNA was inserted into the incised pcDNA3.1-EGFP vector and identified by sequencing. The results of the sequence comparisons were consistent (Figure 2B). Accordingly, the pcDNA3.1-EDN3 plasmid was successfully constructed.

PcDNA3.1-EDN3 and pcDNA3.1-EGFP were transfected into WL poGCs to investigate the effects of *EDN3* overexpression. After transfection for 6 h, fluorescence was observed, indicating that the plasmid was successfully transferred into the cell (Figure 2C). QPCR results demonstrated that the expression level of *EDN3* mRNA was significantly increased after transfection for 6 or 24 h, compared with that empty plasmid (Figure 2D). These results indicated that *EDN3* was successfully overexpressed in GCs.

### 3.3. EDN3 Promotes the Cell Viability and DNA Replication of GCs

**Figure 3:**
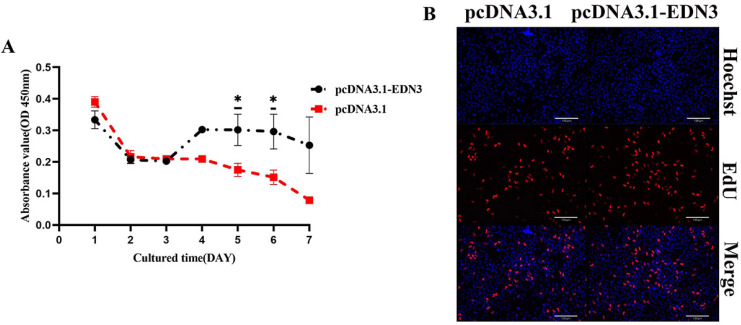
The effect of *EDN3* on the cell viability and DNA replication of GCs in vitro. (**A**) CCK8 detects cell viability after *EDN3* overexpression (six repetitions at each time point). (**B**) Detected DNA replication in GCs. Hoechst stained cell nuclei (top). EdU stained cell indicating DNA replication (middle). The bottom were merged images. Primary WL poGCs were cultured for 6 h with EdU-containing medium in the transfection of pcDNA3.1 and pcDNA3.1-EDN3 (pcDNA3.1 was treated as control). Bar: 130 μm. Data are presented as the mean ± standard deviation (SD). * *p* < 0.05.

GCs were observed using optical microscopy. After 6 h of implantation of recombinant plasmids into GCs, the cells were adherent. The number of cells in the overexpression group was similar to that of the control group. The initial time was depicted as GCs at 0 h of adherence. In the overexpression group, the number of cells increased slightly during the 24 h experimental trial. Microscopic observations revealed that *EDN3* overexpression promoted the cell viability of GCs. Consistent with this, CCK8 analysis depicted that the OD450 value of the *EDN3* overexpression group was significantly higher than that of the empty carrier control group at 5, 6, and 7 d (Figure 3A). The proportion of EdU-positive cells in *EDN3*-overexpressed treatment was higher (18.32% ± 1.63%) than that in the control group (14.95% ± 1.24%). This suggests an increase in DNA synthesis activity associated with *EDN3* overexpression (Figure 3B).

### 3.4. EDN3 Overexpression Gene Affects the Production of Sexual Hormones

**Figure 4:**
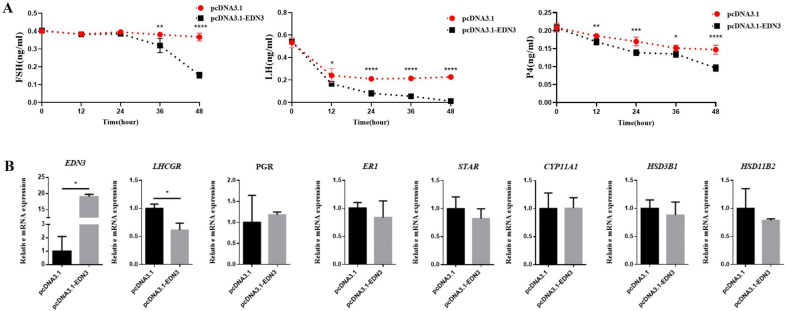
Effect of *EDN3* on gonadal hormone synthesis in GCs. (**A**) ELISA detects the secretion ability of sex hormones (FSH, LH, and P4) after *EDN3* overexpression. Each time point represents the mean ± SEM of at least six replicates. Significant differences between *EDN3* overexpression and control group are indicated with asterisks. * *p* < 0.05, ** *p* < 0.01, *** *p* < 0.001, **** *p* < 0.0001. (**B**) qPCR detects the expression of sex hormone genes by comparing pcDNA3.1 with pcDNA3.1-EDN3 (*n* = 3). Data are presented as the mean ± standard deviation (SD). * *p* < 0.05.

FSH, LH, and P4 secreted by GCs were detected using an ELISA assay. *EDN3* overexpression significantly reduced the ability of GCs to secrete sexual hormones. Most obviously, the LH hormone was reduced the most, and after 48 h, it showed a negligible secretion level (Figure 4A).

Q-PCR results revealed that *EDN3* overexpression could significantly decrease *LHR* expression; however, the expressions of *PGR*, *ER1*, *STAR*, *CYP11A1*, *HSD3B1*, and *HSD11B2* were significantly unaffected. These results indicate that *EDN3* overexpression could inhibit sexual hormone synthesis in GCs, especially LH and *LHR* expressions (Figure 4B).

### 3.5. Differentially Expressed Genes (DEGs) in EDN3-Overexpressed GCs

**Figure 5:**
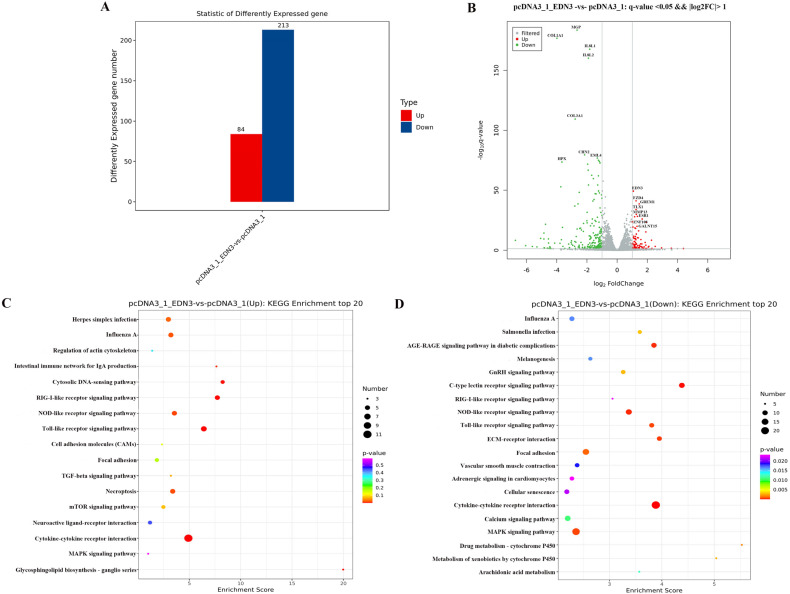
RNA-Seq detected the effect of *EDN3* on GCs. (**A**) Statistical analysis of differentially expressed genes by comparing pcDNA3.1-EDN3 with pcDNA3.1 (*n* = 3). (**B**) Volcano plot of upregulated and downregulated genes. (**C**) Top 20 KEGG pathways of upregulated genes in pcDNA3.1-EDN3 compared with pcDNA3.1. (**D**) Top 20 KEGG pathways of downregulated genes in pcDNA3.1-EDN3 compared with pcDNA3.1. The x-axis represents the enrichment score; the larger the bubble, the greater the number of genes encoding differential proteins. The color of the dots represents the range of the q values.

We detected DEGs in *EDN3*-overexpressed and control cells using transcriptome sequencing. The molecular mechanisms of biological processes can be elucidated through changes in gene expression. In our study, high-throughput transcriptomic analysis of *EDN3* overexpression in WL poGCs identified 297 DEGs (Figure 5A). These data indicated that *EDN3* significantly altered many genes. For example, matrix Gla protein (MGP) is mostly known to be a calcification inhibitor, because its absence leads to delayed bone growth [28]. Decreased expression was also observed in the collagen type I alpha 1 chain gene (*COL1A1*), which confers a survival advantage to the cells [29]. Chimerin 2 (*CHN2*), which encodes GTPase-activating proteins, promotes cell division [30]. The expression levels of numerous genes were also significantly increased. For example, frizzled class receptor 4 (*FZD4*) is a G-protein-coupled receptor that activates diverse intracellular signaling pathways [31]. Similarly, gremlin 1, a DAN family BMP antagonist (*GREM1*) inhibited BMP signaling [32]. A similar reduction in the expression level was also observed in T cell leukemia homeobox 1 (*TLX1*), which can determine cell fate [33] (Figure 5B).

To reveal the functional aspects of these DEGs, 41 gene ontology (GO) analysis terms were summarized for biological processes, cellular components, and molecular functions. A total of 21 GO terms were categorized as biological processes, including cellular processes, immune system processes, and reproduction. Ten were categorized into cellular components, including the extracellular matrix, membrane, and virion, and nine were categorized into molecular functions, including antioxidant, channel regulator, molecular transducer, and transporter activities (Supplementary Tables S1 and S2).

A Kyoto Encyclopedia of Genes and Genomes (KEGG) analysis revealed the potential signaling pathways for the DEGs. KEGG analysis of the differential gene signaling pathways revealed that cytokine–cytokine receptor interaction, rig-i-like receptor signaling pathway, Toll-like receptor signaling pathway, and mucin-type o-glycan biosynthesis pathway were enriched in the *EDN3* overexpression treatment (Figure 5C). The Nod-like receptor signaling pathway, age-range signaling pathway in diabetic complications, and MAPK signaling pathway were downregulated in the overexpression group (Figure 5D).

## 4. Discussion

### 4.1. Multifaceted Role of EDN3 in Melanocyte Proliferation and Reproductive Endocrinology

SF is a natural *EDN3* mutant animal, and the increase in *EDN3* expression level is the reason for black viscera in SF [34]. In vitro studies of quail and mouse embryos have demonstrated that *EDN3* increases melanocytes in a dose-dependent manner [35]. Studies have also shown that, during the differentiation of NCCs into melanoblasts, a large amount of *EDN3* in SF embryos can accelerate the proliferation of melanocyte progenitor cells [12,36]. Our previous study indicated that the egg production rate of purple-crested chickens with the same pedigree was significantly lower than that of red-crested chickens. These selection signals suggest that this difference in egg production is linked to the *EDN3* gene [37]. This suggests that the *EDN3* gene is not only responsible for melanocyte proliferation but may also have multiple functions, including the regulation of reproductive endocrinology.

### 4.2. EDN3 Modulates GC Viability and Hormonal Synthesis via cAMP Signaling Pathway in Reproductive Biology

To study the effect of *EDN3* on the biological functions of GCs, we focused on cell viability and differentiation. *EDN3* can promote GC viability and proliferation, consistent with its role in promoting melanocyte proliferation [12,36]. In exploring the effect of *EDN3* on sexual hormone synthesis in GCs, we found that LH secretion and *LHR* expression significantly declined. LH is essential for follicular development and ovulation [38]. In mammals, LH can stimulate progesterone production, angiogenesis, and trophoblast differentiation during pregnancy [39]. Combining LH and *LHR* can activate the cAMP-PKA pathway, which initiates the expression of a new set of signaling mediators, leading to estrogen production [40].

Following this study approach, we also demonstrated that the *EDN3*-mediated reduction of LH synthesis in GCs occurs through the cAMP signaling pathway. Indeed, the ELISA results depicted that *EDN3* overexpression could inhibit cAMP secretion (Figure S1). Consequently, we performed an association analysis of *EDN3* and LH according to the KEGG signaling pathway and detected changes in the expression of relevant essential genes in the signaling pathway. Similarly, transcriptome sequencing data were used to study the molecular mechanism changes after *EDN3* overexpression. The expression of GPCR-related genes was also significantly altered. These results suggest that the *EDN3* gene may regulate the growth and development of ovarian follicles through G-protein family coupling-related genes.

cAMP promotes extracellular ligand binding to G-protein-coupled receptors, which triggers the catalytic activity of adenylyl cyclases (AC). We examined the expression of AC family genes *ADCY3, ADCY6, ADCY8*, and *ADCY9*. However, the results displayed that although the expression of AC family genes declined, the effect was non-significant (Figure S2).

cAMP activation of PKA signals the nucleus and regulates gene expression. This process occurs through PKA-catalyzed subunit nuclear entry and phosphorylation of transcription factors such as cAMP response element-binding protein (CREB). It binds to cAMP-responsive elements in its target gene promoter, stimulating target gene transcription [40]. Transcriptional induction of cAMP is mediated by the interaction of CREB with the cAMP response element (CRE) in the target gene promoters. In steroid-producing cells, STAR protein activation is regulated by cAMP-mediated signaling. It has been reported that CREB collaboratively regulates cAMP reactivity with other transcription factors [41]. A previous study suggested that *CREB3L1* promotes *STAR* expression [41]. However, in our study, the expression of *CREB3L1* was significantly changed, but there was no significant decline in *STAR* expression (Figure S3). This may be because the expression of CREB regulation *STAR* usually requires synergism with its transcription factors, and the individual effect is non-significant.

In conclusion, we speculate that *EDN3* expression increases the expression of the receptor *EDNRB2*, which leads to increased inhibitory regulation of G protein (Gi). This inhibition likely attenuates cAMP-PKA signaling activation, which impedes *CREB3L1*-mediated promotion of *STAR* expression, subsequently affecting *LHR* expression and LH secretion (Figure S4). To the best of our knowledge, this study represents the first investigation into the direct effects of *EDN3* on the regulation of granulosa cell (GC) biological functions.

### 4.3. EDN3 Influences Gene Expression and Granulosa Cell Differentiation in Development

To further understand the effect of *EDN3* on GCs, DEGs were analyzed using RNA-Seq. *EDN3* promotes differential gene expression related to cellular transcription and embryonic development. *OLIG3, LHX5*, and *NEUROD1* are involved in cellular transcription. *SIX1, SOX2,* and *PAX1* regulate embryonic development. Many DEGs, including *ER*, *GDF6*, and *OSR2*, were associated with ovarian function. These data indicate that *EDN3* could promote GC differentiation. Consistent with the hypothesis that *EDN3* promotes NCC differentiation into different cell types [42].

Numerous genes involved in immune-related functions and G protein coupling were downregulated by *EDN3*. In most human cancers, endothelin activates the MAPK, NF-kB, β-catenin, PI3K/AKT, and Rho GTPase pathways, regulating the expression of genes essential for cell survival, proliferation, drug resistance, angiogenesis, osteogenesis, immune modulation, invasion, and metastasis [43]. Chicken granulosa cells undergo differentiation from prehierarchical to preovulatory GCs via G-protein signaling [44]. Moreover, GO analysis revealed that upregulated entries in the biological processes of cells were mainly concentrated in cell development, DNA transcription, and cell proliferation.

According to the classification of cell components, *EDN3* promotes GC viability and proliferation. Therefore, genes related to cell connections were also upregulated. Molecular functions are enriched in cytokine activity and transcriptional activation. This also indicated that GCs were in a more active proliferative state. However, *EDN3* downregulates the G-protein-coupled receptor signaling pathway in GCs.

### 4.4. Research Limitations and Prospects

Experiment data and transcriptome analyses indicate that *EDN3* inhibits *LHCGR* expression by regulating the cAMP-PKA pathway. To substantiate these findings, we tried to salvage the inhibition effect of *EDN3* by adding forskolin (cAMP-PKA signaling pathway activator). However, post-transfection application of forskolin resulted in cell mortality in primary granulosa cells, suggesting that further investigations are required to elucidate these effects fully.

In mammals, an inverse relationship exists between the proliferative and differentiation potentials of cells: precursor cells typically divide prior to achieving full differentiation, and terminal differentiation generally coincides with the cessation of the proliferation cycle [45]. We also found that the promotion of GC proliferation and inhibition differentiation occurred simultaneously after *EDN3* overexpression in chicken preovulatory follicles. This indicates that *EDN3* may be an important reason for the functional differences in GCs between high- and low-laying hens. This is the first investigation into the role of endothelin in regulating gonadal hormone production in the GCs of hens. Although the ability of endothelin to regulate ovarian sexual hormone production in mammals has been previously investigated, our findings suggest that *EDN3* can block the secretion and expression of LH-LHR in GCs, which may hinder their reproductive ability to some extent. Future studies should explore in more detail the molecular regulatory networks involved in *EDN3* regulation.

## Figures and Tables

**Table 1:** The primers used to detect the targeted genes by RT-PCR.

Primer Name	Primer Sequence	Size (bp)	Temperature
EDN3-F	CTATAGGGAGAGACCCAAGCTGGGCCACCATGGAGCTGGGCTTATTG	522	57 °C
EDN3-R	CCAAGCTTAAGTTTAAACGCTAGGCCTGAGCGAGCGTGTGCTCC		

**Table 2:** The primers used to detect the targeted genes by qPCR.

Genes	Primer Sequence (5′-3′)	Annealing Temperature	Size (bp)
*EDN3*	F-TCAACACCCCAGAGAGGACT R-AGAGCACCGAAATGAAGGCT	59 °C	116
*EDNRB2*	F-CCTCATTGCTCTGCCCATCA R-GCCACTGCTCGATACCTGTC	58 °C	159
*LHCGR*	F- ACGAATCGCTGACACTCAAAC R-CTCTCAGGGCATCGTTGTGT	60 °C	138
*PGR*	F-TAACGCAAAGGCTGTCC R-CCGAAATCCTGGTAGCA	55 °C	215
*ER1*	F-TATTGATGATCGGCTTAGTCTGGC R-CGAGCAGCAGTAGCCAGTAGCA	63 °C	145
*STAR*	F-CCATCAGCCAGGAGCTCAG R-ATCTCGCTGAAGGGCTTCTC	58 °C	124
*CYP11A1*	F-TACCGTGACTACCGCAACAA R-AAAAAGTCCTGGCTCACCTGG	60 °C	152
*HSD3B1*	F-GCCAGAGGATCGTTCGCTTA R-CATCTCGGATGTCCCCTTCC	59 °C	153
*HSD11B2*	F-CACACCAATGGCACAGGTCTC R-GTGCGGAAGTTGCCCAATG	61 °C	98
*ADCY3*	F-CGAGCTGGACAAGTGTGGAT R-AGCTCGTGGCGAGAGAAGTA	60 °C	102
*ADCY6*	F-GCCCGACAGGATTCAGGTAA R-CACCTTAATGACGCCTCGGT	60 °C	88
*ADCY8*	F-CTGGTGGAATTCCTGGGAGA R-CGTTCTTTGCCATGCCCTTC	59 °C	94
*ADCY9*	F-CTGCGATGTCAGCATAGACGA R-TCCTCCTGGAGCCTTCGTA	60 °C	82
*PRKACB*	F-CGAGATCGAGAGCGGCATTCA R-CAAGTTCAGAGACGTGATGGT	60 °C	85
*NFATC1*	F-GCGGCGGAGGAAGAACACTA R-GACAATGAGGAATGCGCCAC	60 °C	83
*CREB3L1*	F-TGAAATGACTCCAGCACCTGT R-CTGCACCAGGTCATCTGC	58 °C	85
*CREB5*	F-AACCTTCCCTCAGTCCAACG R-CTTTGATTCCTCATAAATCGCG	58 °C	123
*CREB3L2*	F-ATGGAGGTGTGCGAGAGC R-AATTCTGTGAAGTGCGTGTGG	59 °C	119
*GAPDH*	F-TCGGAGTCAACGGATTTGGC R-ACAGTGCCCTTGAAGTGTCC	60 °C	163

## Data Availability

The original contributions presented in the study are included in the Supplementary Materials, and further inquiries can be directed to the corresponding author.

## Supplementary Materials

The following supporting information can be downloaded at: https://www.mdpi.com/article/10.3390/cimb46080464/s1.

## References

[1] Yanagisawa M., Kurihara H., Kimura S., Tomobe Y., Kobayashi M., Mitsui Y., Yazaki Y., Goto K., Masaki T. (1988). A novel potent vasoconstrictor peptide produced by vascular endothelial cells. Nature.

[2] Inoue A., Yanagisawa M., Kimura S., Kasuya Y., Miyauchi T., Goto K., Masaki T. (1989). The human endothelin family: Three structurally and pharmacologically distinct isopeptides predicted by three separate genes. Proc. Natl. Acad. Sci. USA.

[3] Davenport A.P., Hyndman K.A., Dhaun N., Southan C., Kohan D.E., Pollock J.S., Pollock D.M., Webb D.J., Maguire J.J. (2016). Endothelin. Pharmacol. Rev..

[4] Bondurand N., Dufour S., Pingault V. (2018). News from the endothelin-3/EDNRB signaling pathway: Role during enteric nervous system development and involvement in neural crest-associated disorders. Dev. Biol..

[5] Clouthier D.E., Hosoda K., Richardson J.A., Williams S.C., Yanagisawa H., Kuwaki T., Kumada M., Hammer R.E., Yanagisawa M. (1998). Cranial and cardiac neural crest defects in endothelin-A receptor-deficient mice. Development.

[6] Kohan D.E., Rossi N.F., Inscho E.W., Pollock D.M. (2011). Regulation of blood pressure and salt homeostasis by endothelin. Physiol. Rev..

[7] Schiffrin E.L. (2018). Does Endothelin-1 Raise or Lower Blood Pressure in Humans?. Nephron.

[8] Ko C., Gieske M.C., Al-Alem L., Hahn Y., Su W., Gong M.C., Iglarz M., Koo Y. (2006). Endothelin-2 in ovarian follicle rupture. Endocrinology.

[9] Palanisamy G.S., Cheon Y.P., Kim J., Kannan A., Li Q., Sato M., Mantena S.R., Sitruk-Ware R.L., Bagchi M.K., Bagchi I.C. (2006). A novel pathway involving progesterone receptor, endothelin-2, and endothelin receptor B controls ovulation in mice. Mol. Endocrinol..

[10] Baynash A.G., Hosoda K., Giaid A., Richardson J.A., Emoto N., Hammer R.E., Yanagisawa M. (1994). Interaction of endothelin-3 with endothelin-B receptor is essential for development of epidermal melanocytes and enteric neurons. Cell.

[11] Lahav R., Ziller C., Dupin E., Le Douarin N.M. (1996). Endothelin 3 promotes neural crest cell proliferation and mediates a vast increase in melanocyte number in culture. Proc. Natl. Acad. Sci. USA.

[12] Lecoin L., Sakurai T., Ngo M.-T., Abe Y., Yanagisawa M., Le Douarin N.M. (1998). Cloning and characterization of a novel endothelin receptor subtype in the avian class. Proc. Natl. Acad. Sci. USA.

[13] Iwai M., Hasegawa M., Taii S., Sagawa N., Nakao K., Imura H., Nakanishi S., Mori T. (1991). Endothelins inhibit luteinization of cultured porcine granulosa cells. Endocrinology.

[14] Calogero A.E., Burrello N., Ossino A.M. (1998). Endothelin (ET)-1 and ET-3 inhibit estrogen and cAMP production by rat granulosa cells in vitro. J. Endocrinol..

[15] Apa R., Miceli F., de Feo D., Mastrandrea M.L., Mancuso S., Napolitano M., Lanzone A. (1998). Endothelin-1 inhibits basal and human chorionic gonadotrophin-stimulated progesterone production. Hum. Reprod..

[16] Klipper E., Levit A., Mastich Y., Berisha B., Schams D., Meidan R. (2010). Induction of endothelin-2 expression by luteinizing hormone and hypoxia: Possible role in bovine corpus luteum formation. Endocrinology.

[17] Choi D.-H., Kim E.K., Kim K.-H., Lee K.-A., Kang D.-W., Kim H.Y., Bridges P., Ko C. (2011). Expression pattern of endothelin system components and localization of smooth muscle cells in the human pre-ovulatory follicle. Hum. Reprod..

[18] Uchide T., Masuda H., Mitsui Y., Saida K. (1999). Gene expression of vasoactive intestinal contractor/endothelin-2 in ovary, uterus and embryo: Comprehensive gene expression profiles of the endothelin ligand-receptor system revealed by semi-quantitative reverse transcription-polymerase chain reaction analysis in adult mouse tissues and during late embryonic development. J. Mol. Endocrinol..

[19] Tai Y., Yang X., Han D., Xu Z., Cai G., Hao J., Zhang B., Deng X. (2022). Transcriptomic diversification of granulosa cells during follicular development between White Leghorn and Silky Fowl hens. Front. Genet..

[20] Gilbert A.B., Evans A.J., Perry M.M., Davidson M.H. (1977). A method for separating the granulosa cells, the basal lamina and the theca of the preovulatory ovarian follicle of the domestic fowl (*Gallus domesticus*). J. Reprod. Fertil..

[21] Chen S., Zhou Y., Chen Y., Gu J. (2018). fastp: An ultra-fast all-in-one FASTQ preprocessor. Bioinformatics.

[22] Kim D., Langmead B., Salzberg S.L. (2015). HISAT: A fast spliced aligner with low memory requirements. Nat. Methods.

[23] Roberts A., Trapnell C., Donaghey J., Rinn J.L., Pachter L. (2011). Improving RNA-Seq expression estimates by correcting for fragment bias. Genome Biol..

[24] Anders S., Pyl P.T., Huber W. (2015). HTSeq—A Python framework to work with high-throughput sequencing data. Bioinformatics.

[25] Love M.I., Huber W., Anders S. (2014). Moderated estimation of fold change and dispersion for RNA-seq data with DESeq2. Genome Biol..

[26] The Gene Ontology Consortium (2019). The Gene Ontology Resource: 20 years and still GOing strong. Nucleic Acids Res..

[27] Kanehisa M., Araki M., Goto S., Hattori M., Hirakawa M., Itoh M., Katayama T., Kawashima S., Okuda S., Tokimatsu T. (2008). KEGG for linking genomes to life and the environment. Nucleic Acids Res..

[28] Laurent C., Marano A., Baldit A., Ferrari M., Perrin J.-C., Perroud O., Bianchi A., Kempf H. (2022). A preliminary study exploring the mechanical properties of normal and Mgp-deficient mouse femurs during early growth. Proc. Inst. Mech. Eng. Part H.

[29] Ma H.-P., Chang H.-L., Bamodu O.A., Yadav V.K., Huang T.-Y., Wu A.T.H., Yeh C.-T., Tsai S.-H., Lee W.-H. (2019). Collagen 1A1 (COL1A1) Is a Reliable Biomarker and Putative Therapeutic Target for Hepatocellular Carcinogenesis and Metastasis. Cancers.

[30] Casado-Medrano V., Barrio-Real L., Gutiérrez-Miranda L., González-Sarmiento R., Velasco E.A., Kazanietz M.G., Caloca M.J. (2020). Identification of a truncated beta1-chimaerin variant that inactivates nuclear Rac1. J. Biol. Chem..

[31] Strakova K., Matricon P., Yokota C., Arthofer E., Bernatik O., Rodriguez D., Arenas E., Carlsson J., Bryja V., Schulte G. (2017). The tyrosine Y250(2.39) in Frizzled 4 defines a conserved motif important for structural integrity of the receptor and recruitment of Disheveled. Cell. Signal..

[32] KKobayashi H., Gieniec K.A., Wright J.A., Wang T., Asai N., Mizutani Y., Lida T., Ando R., Suzuki N., Lannagan T.R. (2021). The Balance of Stromal BMP Signaling Mediated by GREM1 and ISLR Drives Colorectal Carcinogenesis. Gastroenterology.

[33] Cheng L., Arata A., Mizuguchi R., Qian Y., Karunaratne A., A Gray P., Arata S., Shirasawa S., Bouchard M., Luo P. (2004). Tlx3 and Tlx1 are post-mitotic selector genes determining glutamatergic over GABAergic cell fates. Nat. Neurosci..

[34] Dorshorst B., Molin A.-M., Rubin C.-J., Johansson A.M., Strömstedt L., Pham M.-H., Chen C.-F., Hallböök F., Ashwell C., Andersson L. (2011). A complex genomic rearrangement involving the endothelin 3 locus causes dermal hyperpigmentation in the chicken. PLoS Genet..

[35] Hallet M.M., Ferrand R. (1984). Quail melanoblast migration in two breeds of fowl and in their hybrids: Evidence for a dominant genic control of the mesodermal pigment cell pattern through the tissue environment. J. Exp. Zool..

[36] Nataf V., Amemiya A., Yanagisawa M., Le Douarin N.M. (1998). The expression pattern of endothelin 3 in the avian embryo. Mech. Dev..

[37] Dong X., Li J., Zhang Y., Han D., Hua G., Wang J., Deng X., Wu C. (2019). Genomic Analysis Reveals Pleiotropic Alleles at EDN3 and BMP7 Involved in Chicken Comb Color and Egg Production. Front. Genet..

[38] Son W.Y., Das M., Shalom-Paz E., Holzer H. (2011). Mechanisms of follicle selection and development. Minerva Ginecol..

[39] Lonergan P. (2011). Influence of progesterone on oocyte quality and embryo development in cows. Theriogenology.

[40] Weedon-Fekjaer M.S., Tasken K. (2012). Review: Spatiotemporal dynamics of hCG/cAMP signaling and regulation of placental function. Placenta.

[41] Manna P.R., Eubank D.W., Lalli E., Sassone-Corsi P., Stocco D.M. (2003). Transcriptional regulation of the mouse steroidogenic acute regulatory protein gene by the cAMP response-element binding protein and steroidogenic factor 1. J. Mol. Endocrinol..

[42] Square T.A., Jandzik D., Massey J.L., Romášek M., Stein H.P., Hansen A.W., Purkayastha A., Cattell M.V., Medeiros D.M. (2020). Evolution of the endothelin pathway drove neural crest cell diversification. Nature.

[43] Dagamajalu S., Rex D., Gopalakrishnan L., Karthikkeyan G., Gurtoo S., Modi P.K., Mohanty V., Mujeeburahiman M., Soman S., Raju R. (2021). A network map of endothelin mediated signaling pathway. J. Cell Commun. Signal..

[44] Kim D., Johnson A.L. (2018). Differentiation of the granulosa layer from hen prehierarchal follicles associated with follicle-stimulating hormone receptor signaling. Mol. Reprod. Dev..

[45] Ruijtenberg S., van den Heuvel S. (2016). Coordinating cell proliferation and differentiation: Antagonism between cell cycle regulators and cell type-specific gene expression. Cell Cycle.

