# Supplementary material for: Endothelin-3 Suppresses Luteinizing Hormone Receptor Expression by Regulating the cAMP-PKA Pathway in Hen Granulosa Cells
Source: Curr Issues Mol Biol. 2024 Jul 23;46(8):7832–45. doi: 10.3390/cimb46080464 (PMC11352380; doi:10.3390/cimb46080464)
Supplement: Supplementary file 1 [file cimb-46-00464-s001.zip › cimb-3055542-supplementary/cimb-3055542 Supplementary Figures.pdf]

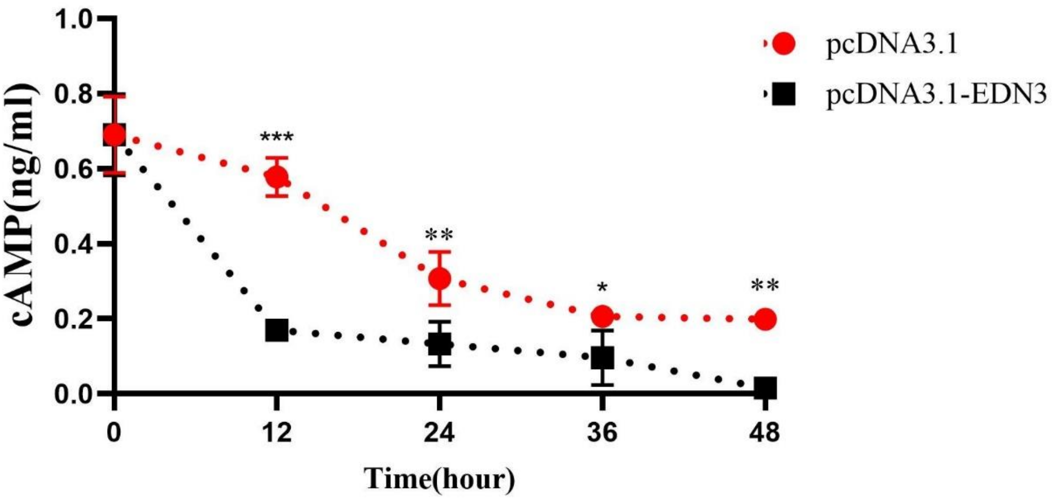

Figure S1. ELISA detected the secretion ability of cAMP after EDN3 overexpression.

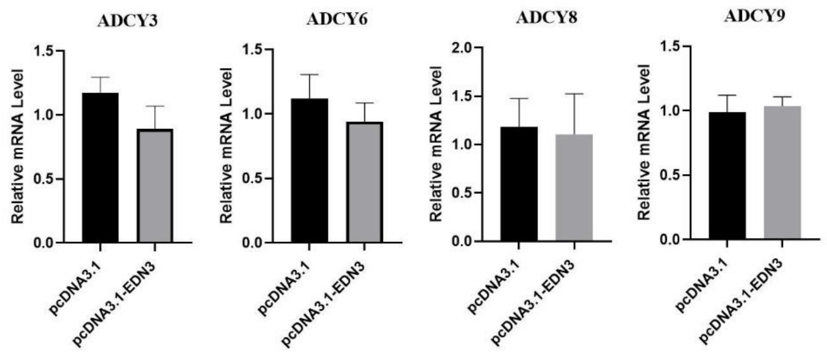

Figure S2. QPCR was used to detect the expression of ADCY family genes.

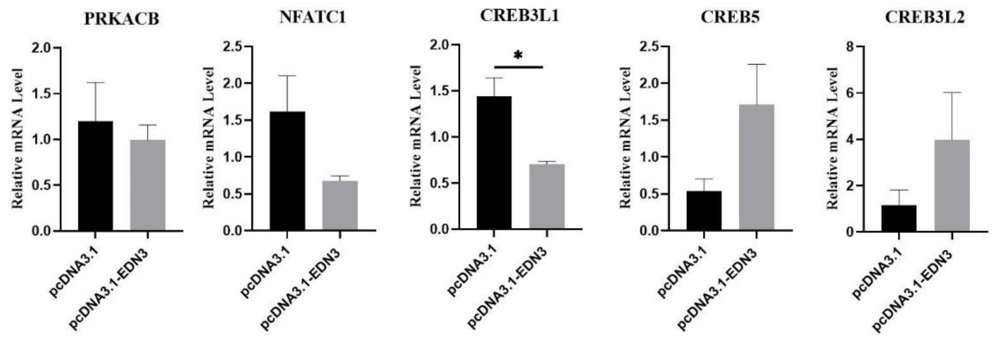

Figure S3. QPCR detected the downstream genes expression of cAMP-PKA signaling pathway.

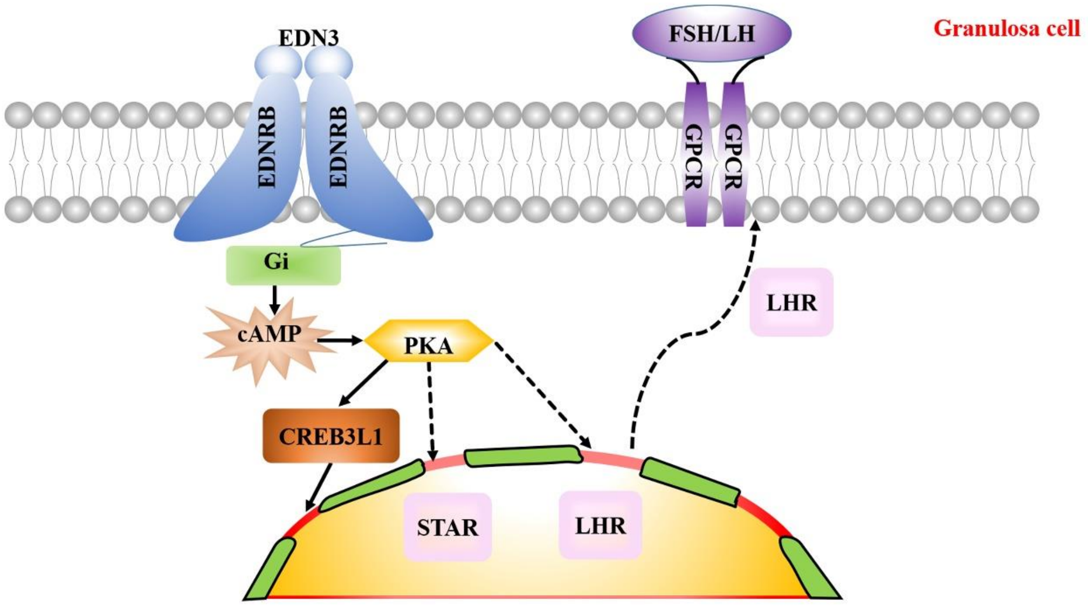

Figure S4. Endothelin-3 suppresses luteinizing hormone secretion by regulating cAMP-PKA pathway
